# An integrated genomic approach identifies that the PI3K/AKT/FOXO pathway is involved in breast cancer tumor initiation

**DOI:** 10.18632/oncotarget.6354

**Published:** 2015-11-22

**Authors:** Linda Smit, Katrien Berns, Katherine Spence, W. David Ryder, Nik Zeps, Mandy Madiredjo, Roderick Beijersbergen, René Bernards, Robert B. Clarke

**Affiliations:** ^1^ Division of Molecular Carcinogenesis and Cancer Genomics Center Netherlands, The Netherlands Cancer Institute, Plesmanlaan, CX, Amsterdam, The Netherlands; ^2^ Breast Biology Group, Breast Cancer Now Research Unit, Institute of Cancer Sciences, University of Manchester, Manchester, UK; ^3^ Department of Medical Statistics, The Christie NHS Trust, Manchester, UK; ^4^ St John of God Subiaco Hospital, Subiaco, Perth, WA, Australia; ^5^ Department of Hematology, VU University Medical Center, Cancer Center Amsterdam, De Boelelaan, Amsterdam, The Netherlands

**Keywords:** breast cancer, stem cells, FOXO, AKT, genetic screen

## Abstract

Therapy resistance is one of the major impediments to successful cancer treatment. In breast cancer, a small subpopulation of cells with stem cell features, named breast cancer stem cells (BCSC), is responsible for metastasis and recurrence of the tumor. BCSC have the unique ability to grow under non-adherent conditions in “mammospheres”. To prevent breast cancer recurrence and metastasis it will be crucial to eradicate BCSC.

We used shRNA genetic screening to identify genes that upon knockdown enhance mammosphere formation in breast cancer cells. By integration of these results with gene expression profiles of mammospheres and NOTCH-activated cells, we identified FOXO3A. Modulation of FOXO3A activity results in a change in mammosphere formation, expression of mammary stem cell markers and breast cancer initiating potential. Importantly, lack of FOXO3A expression in breast cancer patients is associated with increased recurrence rate. Our findings provide evidence for a role for FOXO3A downstream of NOTCH and AKT that may have implications for therapies targeting BCSCs.

## INTRODUCTION

Only a small subpopulation of tumor cells are clonogenic and capable of repopulating a tumor. These tumor-initiating cells have been found to be highly undifferentiated with the capability of both self-renewal and at least partial differentiation. These properties are shared with normal tissue-specific stem cells, hence the name cancer stem cells (CSC), or cancer initiating cells with stem-like properties [1, 2]. It is generally thought that CSC, by virtue of their resistance to therapy, contribute to recurrence and metastasis of tumors [3-5]. CSC were initially identified in leukemia [6] but later also in solid tumors, including the breast, colon and brain [7-10]. A number of developmental signaling pathways such as NOTCH [11, 12], Hedgehog [13], and WNT [14] have been shown to be involved in regulation of self-renewal and differentiation of breast cancer stem and progenitor cells. While the only true marker of breast cancer stem cells (BCSC) is their ability to initiate new tumors, a number of other characteristics are associated with BCSC. For instance, their ability to grow under non-adherent conditions in spheres named “mammospheres” is a property of the tumorigenic subfraction of breast cancer cells [15-17]. Under these mammosphere culture conditions non-stem cancer cells undergo anoikis and there is selection for the CSC-like subpopulation of cells. Mammospheres showed to be capable of *in vivo* tumor formation at limiting dilutions and express high levels of stem cell markers such as OCT4 [15, 17]. Like mammosphere-forming capacity, the ability to exclude Hoechst 33342, expression of CD44, CD24, ESA and CD133, and high aldehyde dehydrogenase (ALDH) activity has been associated with the tumorigenic subfraction of breast cancer [18-23]. Expression of CD133 has also been associated with the chemo sensitivity of breast cancer cells to neoadjuvant therapy [24]. BCSC have been purified from breast cancer patient samples as well as from breast cancer cell lines [7, 12, 16-20, 23].

Increased PI3K/AKT activity correlates with a poor prognosis of breast cancer patients [25, 26] and is described as a major pathway conferring resistance to conventional therapies in multiple tumor types, including breast cancer [25, 27, 28]. Notably, it was shown that the PI3K/AKT pathway, downstream of cytokine and growth factor receptors, contributes to cancer stem cell activity [29]. The FOXO family of transcription factors are major substrates of AKT, which relays PI3K signals to target genes [30]. Inactivation of FOXO3A by the PI3K/AKT pathway favors cell survival, proliferation, and stress sensitivity while activation leads to apoptosis, cell-cycle arrest and stress resistance in most tissues. AKT promotes the inactivation of FOXO3A by its phosphorylation at three serine/threonine residues which leads to the translocation of FOXO3A to the cytoplasm and its targeting for ubiquitination and degradation [30]. Constitutive activation of the PI3K/AKT pathway is a hallmark of many human cancers, including leukemia, breast cancer, glioblastoma and prostate cancer [31, 32].

RNA interference (RNAi) allows suppression of gene expression on a large scale and therewith functional analysis of the role of any gene on specific cellular phenotypes. As such, integration of the results of a RNAi-based genetic screen with gene expression analysis can be used for the unbiased identification of genes that play a causal role in persistence of BCSC. In the present study, we have combined functional genetic approaches with gene expression data and identified FOXO3A as a key player in breast cancer tumor initiation and as such as a potential therapeutic target in breast cancer treatment.

## RESULTS

### A shRNA screen to identify genes that enhance the cancer stem cell phenotype

**Figure 1:**
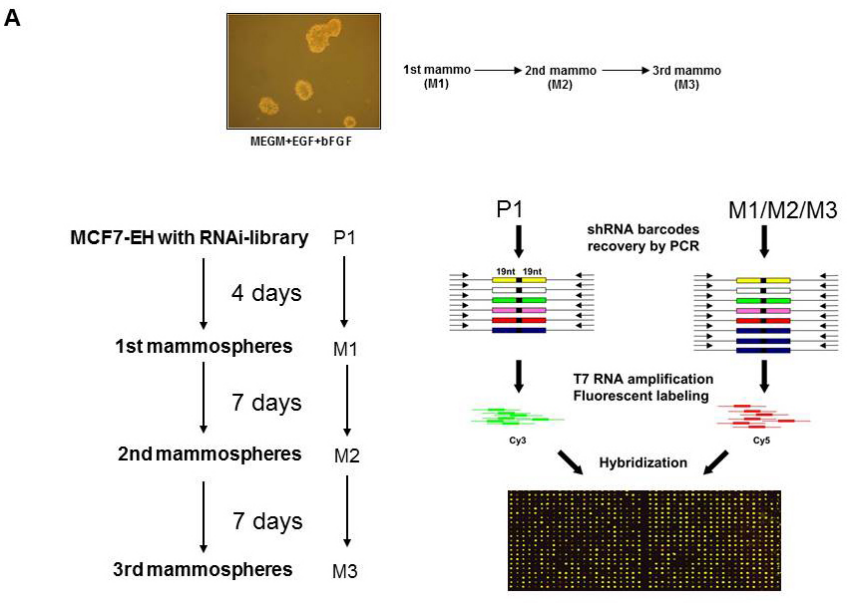

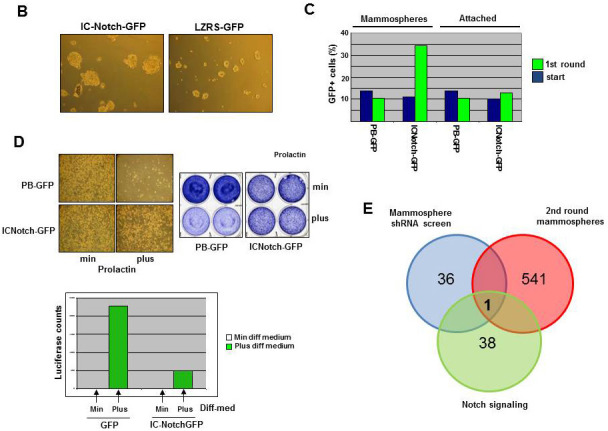
Positive selection shRNA screen mammosphere formation **A.** Schematic illustration of pooled RNAi screen. Cells were transduced with the retroviral NKI library of 24000 shRNAs and part of the cells were used as P1. Cells were cultured for 4 days (M1), 7 days (M2) and 7 days (M3) in mammosphere culture conditions and mammospheres were harvested (M1,M2 and M3). Using PCR amplification, labeling with Cy3 or Cy5 and hybridization to an array containing probes for the 24000 shRNAs present within the MCF7 cells the abundance of each shRNA expression construct in the pool of cells (P1,M1-M3) was determined. NOTCH signaling increases mammosphere formation and blocks differentiation. **B.** Activation of the NOTCH signaling pathway by transduction of IC-NOTCH-GFP in MCF7 cells and the effect on mammosphere formation. **C.** Representative data from one of several experiments showing the increased proportion of GFP-positive cells in mammospheres compared to attached cultures of IC-NOTCH-GFP and control-GFP transduced MCF7 cells. **D.** The effect of expression of IC-NOTCH-GFP or control-GFP on prolactin-induced HC11 cell differentiation shown by a block in proliferation and β-casein promoter activity. A representative β-casein promoter-luciferase reporter expression experiment is shown. **E.** Comparison of the mammosphere shRNA screen, genes downregulated in the 2nd round of mammospheres and genes downregulated in MCF7 cells activated by NOTCH signaling resulted in one gene; FOXO3A.

MCF7 cells can be used in an *in vitro* system in which primitive mammary cancer stem/progenitor cells can be propagated in culture as floating spherical colonies termed “mammospheres”. Mammospheres contain a small number of breast cancer stem cells capable of self-renewal, as well as multipotent progenitors that constitute the tumorigenic MCF7 subfraction [15-17]. We have used an unbiased functional genetic approach to identify shRNAs that enhance growth of MCF7 cells in mammosphere culture using our library of 24,000 shRNAs targeting 8,000 human genes [33]. We infected MCF7 cells with this retroviral shRNA library (P1) and cultured them in mammosphere culture conditions for four days (M1). Single cells suspensions generated from the first round of mammospheres were replated in a second round of mammosphere culture (7 days, M2). Likewise, dissociation of M2 mammospheres and replating in a third mammosphere round was performed (7 days, M3) (Figure 1A). This resulted in four populations of cells (library-infected parental MCF7:P1, and three mammosphere cultured populations: M1-M3). From these populations, shRNAs were recovered by a PCR-based strategy and “bar code” hybridization was performed to measure relative abundance of each of the 24,000 shRNA vectors in the different cell populations as described previously (Figure 1A, outline of the experiment) [28, 33]. Comparison of shRNAs derived from the mammosphere cultures (M1, M2, M3) to shRNAs derived from the original parental cells (P1), identified 36 shRNAs that were more than two fold enriched in MCF7 cells grown in all three mammosphere cultures (M1-M3, Supplemental Table 1, *p* < 0.005 and A>7). Seventeen of these genes were progressively enriched in each subsequent round of mammosphere selection (Table S1, red). The identified shRNAs target genes involved in signaling pathways known to regulate breast cancer stem cells, including WNT (SFRP1), CD44 (FKBPL), and the PI3K/AKT (FOXO3A) signaling pathways [14, 19, 34-36].

### Identification of genes that are decreased in expression in mammospheres

To further prioritize from this list of candidate genes, we searched for genes that are downregulated when MCF7 cells are grown in mammosphere culture. We reasoned that genes whose decrease is causally involved in mammosphere growth should at least be downregulated under these growth conditions. We performed microarray analysis of RNA from mammosphere cultures (first and second round) of MCF7 cells and compared it to RNA from MCF7 cells grown under attached conditions. Some 381 genes were significantly downregulated in the first mammosphere culture (Tables S2A and S1). Mammospheres are enriched in undifferentiated cells indicated by lower expression of cytokeratins 8, 12, 16, 18 and 19 (Table S2B). Among the 36 shRNAs we identified in our mammosphere screen five shRNAs target genes downregulated in the 1^st^ round of mammospheres (HMGB2, FOXO3A, HSPA1B, CD47 and HMG1L10, dark blue boxes in Table S1). Some 541 genes were significantly decreased in expression in the second mammosphere culture (Tables S2C and S1). Also in this round, mammospheres are enriched in undifferentiated cells indicated by the lower expression of several cytokeratins (Table S2D). From the 36 shRNAs we identified in the mammosphere screen, three genes were lower expressed in the second round of mammosphere culture compared to attached MCF7 cells (FOXO3A, HSPA1B, and MPP2, light blue boxes in Table S1). Thus, this combined analysis of a functional screen and expression analysis implicates both FOXO3A and HSPA1B in mammosphere formation.

### Combining a NOTCH-induced gene expression profile with the mammosphere shRNA screen

As a second independent approach to prioritize the genes from the shRNA screen, we took advantage of the fact that NOTCH activation can promote the self-renewal and proliferation of mammary stem/progenitor cells [11, 12]. As previously reported, expression of constitutively active NOTCH (the intracellular domain of Notch, IC-NOTCH-GFP) in MCF7 cells strongly promoted the ability to grow in mammospheres (Figure 1B ), and thus alters stem/progenitor properties of MCF7 cells. An increase in the number of GFP-positive cells was observed in the population that formed mammospheres, whereas cells grown under attached conditions did not result in an increased fraction of GFP positive cells, ruling out the possibility that IC-NOTCH simply conferred a growth advantage to MCF7 cells (Figure 1C). IC-NOTCH also inhibits the differentiation of HC11 cells following the addition of prolactin as shown by a block in cell proliferation and milk protein promoter activity (Figure 1D). We hypothesize that genes downregulated by NOTCH signaling are likely candidates to be causally involved in the ability to grow in mammospheres. We performed microarray analysis on RNA extracted from MCF7 cells expressing IC-NOTCH and from control cells, both cultured under attached conditions, and determined which genes were downregulated by expression of IC-NOTCH (Table S3). Combining the results from the shRNA screen, the gene expression profiling of the 1^st^ and 2^nd^ round of mammospheres and the gene expression profiling of NOTCH-activated MCF7 cells resulted in identification of a single candidate, FOXO3A (Figure 1E). The shRNA against *FOXO3A* was enriched in the genetic screen (increased in every round of mammosphere culture), *FOXO3A* was downregulated in MCF7 mammospheres compared to attached cells and reduced by active NOTCH signaling.

### Modulated FOXO3A activity results in a change in mammosphere number

**Figure 2:**
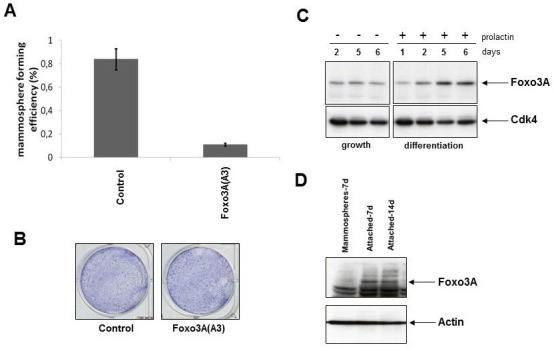
Modification of FOXO3A activity results in a change in number of mammospheres **A.** Mammosphere-forming efficiency is shown for MCF7 cells expressing control or FOXO3A(A3) vectors. Means +/− SEM are shown. *P* < 0.001 by Mann-Whitney U test. **B.** MCF7 cells with FOXO3A(A3) cultured under attached culture conditions. **C.** The induction of differentiation of HC11 cells by prolactin and immunoblotting for the presence of FOXO3A. Cdk4 immunoblotting is used as loading control. **D.** 1th round mammospheres were cultured for 7 days under mammosphere culture conditions or cultured under attached conditions for 7 or 14 days. Immunoblotting for actin is used as a loading control.

The results described above suggest that inhibition of FOXO3A might increase the mammosphere phenotype while activation of FOXO3A might decrease the number of mammospheres. To study the effect of FOXO3A activation on mammosphere formation we used a mutant version of FOXO3A in which the three AKT phosphorylation sites in FOXO3A were mutated to alanines [FOXO3A(A3)], resulting in its retention in the nucleus and constitutive transcriptional activation [37, 38]. Activation of FOXO3A in MCF7 cells through the expression of FOXO3A(A3) leads to a decreased number of mammospheres (Figure 2A), while it did not have an effect on growth of MCF7 cells under attached conditions (Figure 2B).

### FOXO3A is upregulated after differentiation of breast cells

We reasoned that genes whose suppression can cause an increase in mammosphere growth might be increased upon differentiation. We analyzed FOXO3A levels during differentiation of the normal breast cell line HC11 and found that prolactin-induced differentiation resulted in an increase in expression of FOXO3A (Figure 2C). Since mammosphere cultures are enriched for undifferentiated breast cancer cells, culturing of mammospheres under attached conditions induces (partial) differentiation. Culturing of MCF7 mammospheres for 7 or 14 days under attached conditions resulted in an increase in FOXO3A (Figure 2D), consistent with a role for FOXO3A in regulating the differentiation state of MCF7 cells.

### Decreased FOXO3A is associated with BCSC features; increase in CD24^low/min^ cells and CD133 expression

Tumor-initiating breast cancer cells can be distinguished from the non-tumorigenic cancer cells based on presence of the CD44^+^CD24^−/low^ phenotype. To determine whether downregulation of FOXO3A has an effect on number of cells expressing CD24, we looked by flow cytometry at MCF7 cells transduced with a shRNA (shRNA4) against *FOXO3A*. FOXO3A downregulation (Figure S1) resulted in an increase in cells with low (1.17% to 3.15%) and intermediate (0.29% to 1.86%) CD24 expression (Figure S2). In addition to the CD44^high^CD24^low^ phenotype, enhanced CD133 expression has been described to be associated with BCSCs [20, 23]. We downregulated FOXO3A expression by shRNAs in CD133^low^ MCF10A cells (Figure S3) and found that, like active NOTCH signaling (Figure S4A), knockdown of FOXO3A results in an increase in expression of CD133 on the cell membrane (Figure S4B).

### Downregulation of FOXO3A results in modulation of stem cell genes

We generated gene expression profiles from normal breast epithelial cells (MCF10A) and breast cancer cells (MCF7) with knockdown of FOXO3A, by two independent shRNAs against FOXO3A, to identify genes regulated by FOXO3A and possibly involved in regulation of the breast (cancer) stem cell phenotype. Decreased expression of FOXO3A in MCF10A cells results in modulation of the expression of several genes known to be involved in (cancer) stem/progenitor cell function (Tables S4A; upregulated genes and S4B; downregulated genes). Besides the known FOXO3A target gene, growth arrest and DNA damage-inducible gene GADD45A, FOXO3A knockdown affects several signaling proteins involved in enhancing the stem cell-like phenotype. These include upregulation of Ly6E (Sca-1), ID2 and GATA3 (Table S4A) and downregulation of several keratins, IGFBP7 and TIMP3 (Table S4B). Pathway analysis using DAVID indicated that genes up- and down-regulated by FOXO3A knockdown in MCF10A cells are involved in cancer signaling pathways such as the catenin, HIFα and p53 signaling pathway. By gene ontology analysis we showed that genes modified by FOXO3A knockdown are involved in cell proliferation, cell adhesion and regulation of cell death (Table S4C). Like in MCF10A cells, FOXO3A knockdown in MCF7 cells targets the GADD45A gene and affects several signaling pathways that play a role in a stem cell phenotype (Tables S5A; upregulated genes and S5B; downregulated genes), including WNT signaling components (DKK1 and FRAT1), BMP7 signaling and the p53 signaling pathway. By pathway analysis using DAVID (gene ontology analysis) we identified that knockdown of FOXO3A in MCF7 cells affects genes involved in the response to stress and organic substances and the regulation of cell death and proliferation (Table S5C).

### Paclitaxel treatment selects for breast cancer cells with a stem cell phenotype and low FOXO3A expression

**Figure 3:**
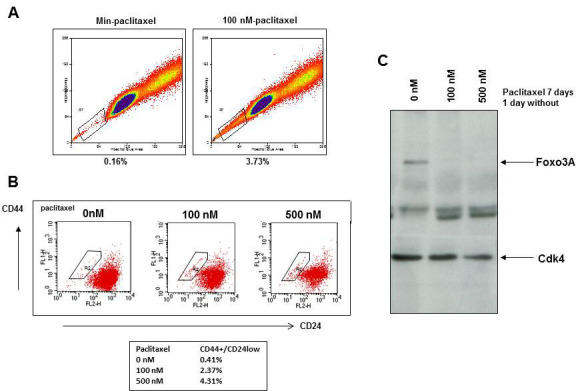
Paclitaxel treatment selects for breast cancer cells with a stem cell phenotype and low FOXO3A expression **A.** MCF7 cells were treated for 24 hr with 100 nM paclitaxel and labeled with Hoechst 33342 for 2 hours. The Hoechst dye was excited with a 405-nm violet laser and detected with 450/BP50 optical filters. **B.** SKBR3 cells were incubated with 100 or 500 nM paclitaxel for 7 days and stained with anti-CD24-Fitc and anti-CD44-PE. **C.** MCF7 cells were incubated with 100 and 500 nM paclitaxel for 7 days and viable cells were lysed and immunoblotted for FOXO3A. CDK4 was used as a loading control.

BCSC are proposed to be less sensitive to therapy and therefore treatment of MCF7 cells with paclitaxel might result in survival of a small subpopulation of cells with stem cell features [39]. Indeed, paclitaxel treatment of MCF7 cells for 7 days resulted in an increase in the so called “side population” of cells that exclude the dye Hoechst 33342 (Figure 3A) and results in a ten-fold increase in the CD44+CD24^low^ population of SKBR3 (Figure 3B) and MCF7 breast cancer cells (Figure S5A). Mammospheres were less sensitive to paclitaxel than MCF7 cells grown in attached conditions (Figure S5B). MCF7 cells surviving 7 days of paclitaxel treatment and therefore resistant to treatment had decreased FOXO3A expression (Figure 3C), indicating a survival advantage for breast cancer cells with low FOXO3A expression after treatment with paclitaxel.

### Active AKT results in enhanced mammosphere formation and therapy resistance

**Figure 4:**
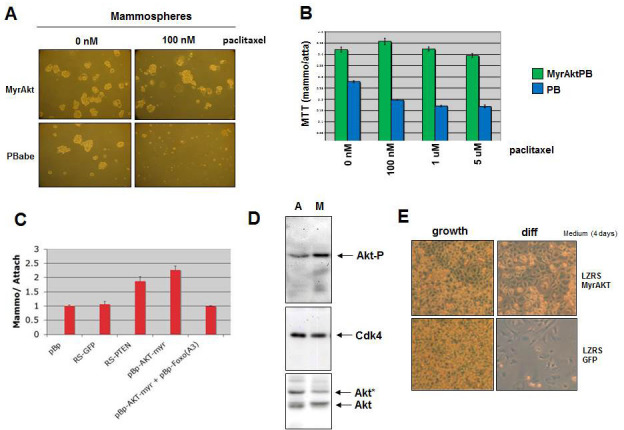
Activated AKT results in increased mammosphere formation, reduced sensitivity to paclitaxel and inhibition of differentiation **A.** MCF7 cells transduced with Myr-AKT were cultured under mammosphere culture conditions in the absence or presence of 100 nM paclitaxel for 7 days. **B.** MCF7 cells with Myr-AKT or control PBabe were cultured under mammosphere and attached conditions in the presence of various concentrations of paclitaxel and an MTT assay was performed. **C.** The bar graph depicts the ratio of MTT values for mammosphere versus attached cultures of MCF7 cells. The cells were transduced with shRNAs for GFP and PTEN or with overexpression of Myr-AKT and Myr-AKT together with mutant FOXO3A(A3) or with solely the mutant FOXO3A(A3) were cultured under mammosphere and attached conditions and an MTT of both was performed. **D.** MCF7 cells were cultured for 7 days under mammosphere and attached conditions, cells were lysed and immunoblotting was performed with anti-phospho AKT, anti-AKT and anti-CDK4. E) HC11 cells transduced with Myr-AKT or control plasmid were induced to differentiate by prolactin, as described for Figure 1D.

FOXO3A is an important downstream target of the PI3K-AKT pathway, transducing signals in response to growth factor stimulation. To determine whether activated AKT can also induce a cancer stem/progenitor cell phenotype in breast cancer cells, we cultured MCF7 cells overexpressing a constitutively activated AKT in mammosphere culturing conditions. The number of mammospheres formed by MCF7 cells with activated AKT is increased compared to MCF7 control cells (Figure 4A and 4B). Activated AKT did not result in enhanced proliferation of MCF7 cells under attached conditions (data not shown). The induction of mammospheres by active AKT could be inhibited by overexpression of FOXO3A(A3)(Figure 4C), indicating that signaling to FOXO3A is responsible for the increase in mammospheres by active AKT. Mammospheres with activated AKT were more resistant to paclitaxel than those without activated AKT (Figure 4A and 4B).

Since mammosphere cultures enrich for breast cancer cells that are in an undifferentiated state we hypothesized that mammospheres might have higher levels of activated AKT than MCF7 cells cultured under attached conditions. Indeed, assessment of the activation of the PI3K/AKT/FOXO pathway by immunoblotting with phospho-specific AKT antibodies revealed an increase in phosphorylated Ser^473^-AKT in mammospheres (Figure 4D).

Since we previously observed that active NOTCH signaling enhances mammosphere formation, increases CD133 expression and induced a block in prolactin driven differentiation of HC11 cells, we determined whether active AKT in the form of myristylated AKT could also, besides inducing mammospheres, block differentiation. Indeed, like active NOTCH signaling, the activation of AKT resulted in a block in prolactin induced differentiation of HC11 cells (Figure 4E) and enhanced expression of CD133 (data not shown).

### FOXO3A activity results in decreased breast cancer initiating potential

**Figure 5:**
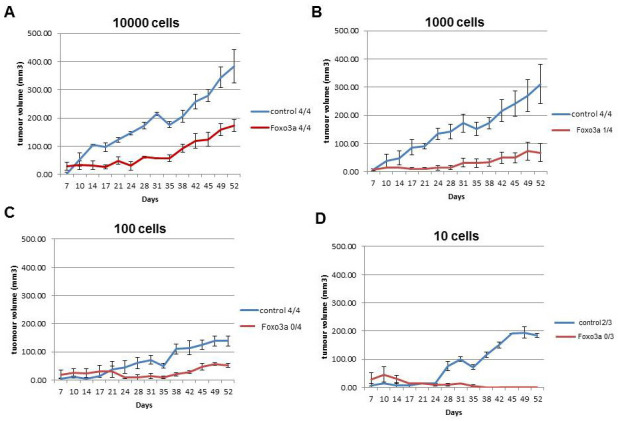
Activation of FOXO3A results in reduced breast cancer intitiating potential **A.** 10000 **B.** 1000 **C.** 100 and **D.** 10 MCF cells transduced with FOXO3A(A3) or the control plasmid were diluted in 0.2ml 50/50 mammosphere medium/matrigel before sub-cutaneous injection into opposing flanks of NSG mice. Tumor-take and growth was monitored every 3 days and tumor volume was calculated in mm^3^. At 52 days, the number of tumors >100mm3 was used as evidence of tumor initiation, and the proportion of tumors >100mm3 out of total injections is shown.

If decreased FOXO3A is partly responsible for preserving the stem cell phenotype of BCSC, enhanced FOXO3A activity might result in a decrease in breast cancer initiating potential of breast cancer cells. To show the effect of FOXO3A on breast cancer initiating potential we subcutaneously injected various numbers of MCF7 cells expressing FOXO3A(A3) into the NOD/SCID IL2 receptor gamma chain knockout (NSG) mice. MCF7 cells with FOXO3A(A3) expression have decreased mammosphere forming capacity (Figure 2A) and lose the potential to initiate a tumor defined by growth to >100 mm^3^ by 50 days (Figure 5A-5D). The subcutaneous injection of 10 control MCF7 cells results in a subcutaneously growing tumor while the injection of 10 MCF7 cells expressing FOXO3A(A3) did not result in tumors above 50 mm^3^ (Figure 5D). A subcutaneous tumor of >100 mm^3^ is reached in fourteen of the fifteen mice injected with the control MCF7 cells while in mice injected with MCF7 cells expressing FOXO3A(A3) tumors >100 mm^3^ is only achieved in five of the fifteen mice (Table 1A). Calculation of the number of breast cancer cells with stem cell (tumor initiating) capacity by the Extreme Limiting Dilution Analysis (ELDA) method using R showed that on average 1 out of 10 MCF7 cells have the potential to initiate a tumor >100mm^3^ while overexpression of FOXO3A(A3) resulted in a ∼300 fold decrease in breast cancer initiating cell frequency (1 into 2778 MCF7 cells) (Table 1B).

**Table 1:** MCF7 tumour-intiating cell frequency is reduced after activation of FOXO3AAB

A			
Transduced MCF7 cells	Cell number injected	Number of mice	Number of tumours >100 mm3
**Control**	**10000**	**4**	**4**
**Control**	**1000**	**4**	**4**
**Control**	**100**	**4**	**4**
**Control**	**10**	**3**	**2**
**Foxo3A(A3)**	**10000**	**4**	**4**
**Foxo3A(A3)**	**1000**	**4**	**1**
**Foxo3A(A3)**	**100**	**4**	**0**
**Foxo3A(A3)**	**10**	**3**	**0**

B				
Transduced MCF7 cells	Lower CI	TIC frequency	Upper CI	Chi-sqd & *P* value
**Control**	**1/39**	**1/9.6**	**1/3**	**Chi-sqd = 35.7**
**Foxo3A(A3)**	**1/8952**	**1/2778**	**1/862**	**P < 0.0001**

(A) The numbers of cells injected, numbers of mice and those positive for tumor growth are shown for control versus FOXO3A (3A)-transduced MCF7 cells. (B) Tumour-initiating cell (TIC) frequency and 95% confidence intervals (CI) demonstrating the highly significant difference in TICs using the Chi-sqd to test the data in A.

### Breast cancer patients with neither FOXO3A in nucleus nor cytoplasm have higher recurrence rate than patients with FOXO3A expression

**Figure 6:**
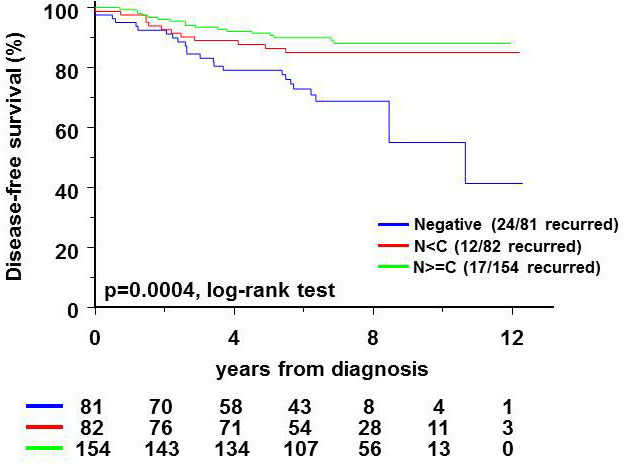
Relationship of FOXO3A expression to recurrence of invasive breast cancer (see Supplemental Table 6 for characteristics) is represented in a Kaplan Meier plot The log-rank test was used to assess the prognostic value of FOXO3A after adjustment for ER and lymph node status. The proportion of events per number of patients for each category is indicated in the figure. N<C - nuclear less than cytoplasmic FOXO3A staining intensity; N=>C - nuclear greater than or equal to cytoplasmic FOXO3A staining intensity.

Our data suggest that downregulation of FOXO3A in breast cancer cells is associated with enhanced stem cell features, including therapy resistance and breast cancer initiating potential. Breast cancer patients with a lack of expression of FOXO3A might therefore have a more rapid recurrence of the disease than patients with FOXO3A expression. Indeed, staining for expression of FOXO3A in tissue microarrays of 317 breast cancer patients (characteristics in Table S6) showed that the ∼25% of patients without FOXO3A have a higher recurrence rate in up to 12 years of follow-up (Figure 6). FOXO3A is phosphorylated by AKT and subsequently degraded in the cytoplasm. Therefore, we also quantified the level of FOXO3A expression in the nucleus and the cytoplasm (Figure S6) and showed that loss of Foxo3A expression correlates with a high recurrence rate (*p* = 0.0004 by log-rank test in multivariate analysis). Differential FOXO3A expression between nucleus or cytoplasm had no influence on recurrence rate (Figure 6).

## DISCUSSION

A body of evidence indicates that a subpopulation of breast cancer cells with stem cell-like features, BCSC, are relatively resistant to chemo-, radio- and endocrine therapies [40-42] and drive tumor recurrence and metastasis [7, 19, 43]. The identification of therapeutic strategies to eliminate these BCSC populations is crucial for improvement of breast cancer outcome. In this study, we describe an integrated approach to search for genes that regulate the BCSC phenotype. By combining a mammosphere shRNA screening approach with identification of genes decreased in mammospheres and NOTCH-activated breast cancer cells we found that suppression of *FOXO3A* increases the number of BCSC, indicating NOTCH/AKT/FOXO3A signaling as a potential therapeutic target for breast cancer treatment. FOXO transcription factors belong to the class of winged helix group of transcription factors and have been implicated in the control of genes involved in multiple cellular processes, including the cell cycle stage, apoptosis, epithelial-to-mesenchymal (EMT) transition, longevity, metabolism and protection from oxidative stress [30]. Some of these cellular processes showed to be associated with CSC properties, including quiescence, EMT and protection from stress. There are a limited number of reports on involvement of FOXO3A in stem cell-like cancer cells. Findings in prostate cancer and glioblastoma [44, 45], and very recently in ovarian, liver and colorectal cancer [46-48] are in line with our identified function of FOXO3A in breast cancer stem cells. In prostate cancer, knockdown of FOXO3A led to expansion of the CSC population as well as to increased self-renewal and tumorigenic capacity [44]. In glioblastoma, decreased FOXO3A increases glioblastoma CSC features [45] and the expression of FOXO3A in human glioma samples is correlated with their malignant grade.

In contrast to our findings, several studies showed that enhanced FOXO3A activity enhances CSC and poor prognosis. In chronic myeloid leukemia, FOXO3A deficiency impaired leukemia-initiating potential [49] and FOXO3A is required for the maintenance of neural stem cells in mice [50]. In acute myeloid leukemia, high expression of FOXO3A is associated with a poor prognosis [51]. Thus, findings about the role of FOXO3A in CSC maintenance are contrasting, suggesting that FOXO3A function is highly dependent on the cancer type, the cell cycle stage of CSC or the possible signaling routes leading to its activation.

FOXO3A expression is decreased in both first and second round of MCF7 mammosphere cultures (compared to attached MCF7 cells) as well as in paclitaxel resistant MCF7 cells, suggesting that immature MCF7 cells are more paclitaxel resistant and have less FOXO3A expression. Like in glioma CSC [45], we observed an increase in FOXO3A expression upon differentiation of both normal breast cells and BCSC and we showed that forced activation of FOXO3A is sufficient to block mammosphere formation and, most importantly, to repress breast cancer initiating potential *in vivo*. Consistent with these results we show that a lack of expression of FOXO3A in breast cancer is associated with a more rapid recurrence rate of the tumor. In contrast to an earlier report on FOXO3A expression and prognosis [34], we did not find that enhanced expression in either nucleus or cytoplasm had an effect on recurrence time.

FOXO3A is a downstream target of the PI3K-AKT pathway and activation of AKT results also in an increase in BCSC features such as enhanced mammosphere formation, inhibition of differentiation and an increase in CD133 expression. In addition to its effect on the stem/progenitor population we show that AKT plays a role in paclitaxel resistance. The induction of mammospheres by activated AKT can be inhibited by FOXO3A activation, showing that FOXO acts downstream of AKT in BCSC signaling.

Efficient targeting of FOXO3A might be possible by inhibition of receptor activation, as the AKT/FOXO3A signaling pathway is activated by many transmembrane receptors, including tyrosine kinase and cytokine receptors [30]. An example of depletion of BCSC by activation of FOXO3A is blocking of the IL-8 receptor CXCR1 by a specific CXCR1 blocking antibody or by repertaxin [52]. Modulation of other membrane receptors that signal through FOXO3A might have similar effects. An example is the recent study by Prabhu et al. in where overexpression of AKT and knockdown of FOXO3A can rescue TRAIL pathway-mediated cell death of CD44 positive colon CSC as well as colonosphere inhibition [47].

Based on our findings we suggest that downregulation of FOXO3A, either by activation of AKT or by other means, results in increased stem cells properties in breast cancer cells and consequently in therapy resistance. Application of PI3K or AKT inhibitors might deplete therapy resistant low expressing FOXO3A breast cancer cells in addition to breast cancer cells with activated PI3K/AKT/β-catenin [35] within a heterogeneous breast cancer cell population. While speculative, these data may also explain why the combination of trastuzumab and chemotherapy shows such strong synergy in HER2-positive breast cancer [53, 54]. Our data would support the notion that trastuzumab reduces AKT signaling to FOXOs, thereby reducing the number of chemotherapy-resistant breast cancer cells in the tumor.

## MATERIALS AND METHODS

### Plasmids

For expression of mutant FoxO the plasmid pcDNA-FoxO3a.A3 was used [Medema et al., 2000]. The wild-type and mutant AKT, and mutant Foxo plasmids were a kind gift of Prof. B. Burgering (UMCU, Utrecht, The Netherlands). FOXO3A was downregulated by retroviral transduction of the plasmid Pretrosuper [Berns et al., 2004 and 2007] with the following hybridized oligonucleotides ligated in:

FOX1FW-5′-GATCCCCCCTGTCCTACGCGGACCTGTTCAAGAGACAGGTCCGCGTAGGACAGGTTTTTGGAA-3′ FOX1RV-5′AGCTTTTCCAAAAACCTGTCCTACGCGGACCTGTCTCTTGAACAGGTCCGCGTAGGACAGGGGG-3′, FOX2FW-5′-GATCCCCATAGCAACAAGTATACCAATTCAAGAGATTGGTATACTTGTTGCTATTTTTTGGAAA-3′, FOX2RV-5′-AGCTTTTCCAAAAAATAGCAACAAGTATACCAATCTCTTGAATTGGTATACTTGTTGCTATGGG-3′, FOX3FW-5′-GATCCCCAGAACTTGCTCCACCACCATTCCAAGAGATGGTGGTGGAGCAAGTTCTTTTTTGGAAA-3′ FOX3RV-5′AGCTTTTCCAAAAAGAACTTGCTCCACCACCATCTCTTGAATGGTGGTGGAGCAAGTTCTGGG-3′, FOX4FW-5′-GATCCCCGTCAGCCAGTCTATGCAAATTCAAGAGATTTGCATAGACTGGCTGACTTTTTTGGAAA-3′, FOX4RV-5′-AGCTTTTCCAAAAAGTCAGCCAGTCTATGCAAATCTCTTGAATTTGCATAGACTGGCTGACGGG-3′, IC-Notch was in the LZRS retroviral plasmid and a kind gift of Dr. B. Blom (Academic Medical Center, Amsterdam, The Netherlands).

### Mammosphere culture

MCF7 and SKBR3 cells were purchased from the American Type Culture Collection (ATCC, Manassas, VA) and cultured in Dulbecco modified medium (DMEM) (Life Technologies, Darmstadt, Germany) supplemented with 10% fetal calf serum (FSC) and penicillin and streptomycin (P/S). The MCF10A cells were cultured in DMEM: Ham's F12 medium (Life Technologies)(1:1) supplemented with 5 μg/ml insulin (Sigma Aldrich, Taufkirchen, Germany), epidermal growth factor (Sigma Aldrich), 1 μg/ml hydrocortisone (Sigma Aldrich), cholera toxin, P/S and 5% horse serum. For mammosphere culture, cells were plated at 1 × 10^4^ cells/cm^2^ in an ultra low-attachment culture plate (Corning, Kaiserslautern, Germany) and cultured in Mammocult medium (Stem Cell Technologies, Grenoble, France) supplemented with human recombinant EGF (20 ng/μl), human recombinant bFGF, 4 μg/ml, insulin, and B27. After culturing for the days indicated, mammospheres were counted, pictures were taken and mammosphere abundance was quantified with MTT assay. MTT (3-[4,5-dimethylthiazol-2-yl]-2,5- diphenyltetrazolium bromide; thiazolyl blue)(Sigma Aldrich) was added and incubated with the cells for 4 hours. MTT crystals were dissolved in isopropanol-HCl. Color conversion was measured at 570 nm and corrected for background at 690 nm.

For generation of second and third round of mammospheres the first/second round of mammospheres were collected, incubated with trypsine and made single cell by resuspending with a syringe with needle and filtered through a 40 um cell strainer (Becton Dickenson, Heidelberg, Germany). MCF7 cells derived from 1^st^ round of mammospheres were plated at 1 × 10^3^/cm2 and cultured again in ultra low-attachment plates in Mammocult medium.

### shRNA screening

MCF7 cells were infected with retroviruses representing the complete NKI shRNA library [Berns et al., 2004 and 2007], selected with puromycin (2 μg/ml) and plated on low attachment culture plates (Corning). Part of the transduced cell populations was taken apart (P1). After four days of culturing on low-attachment plates mammospheres were taken, dissociated into single cells and again cultured in mammosphere culture conditions. This 2^nd^ round of mammospheres was again dissociated and cultured in mammosphere culture condition. Populations of cells were collected and DNA was isolated with the use of DNAzol (Life Technologies). The shRNA inserts were amplified from genomic DNA by PCR. with Expand Long Template PCR system (Roche) and the use of pRS-fw-primer: 5′-GAGACGTGCTACTTCCATTTGTC-3′ and pRS-rev primer: 5′-GAGACGTGCTACTTCCATTTGTC-3′. Purified PCR products were used for linear RNA amplification and purified RNA probes were labeled with cyanine-3 (Cy3) or cyanine-5 (Cy5) fluorescent groups (Kreatech, Eindhoven, The Netherlands). Labeled RNA probes from the various cell populations (P with M1, P with M2 and P with M3) were combined and hybridized to oligonucleotide arrays as described [28]. Quantification of the resulting fluorescent images was performed with Imagene 5.6 (Biodiscovery, Hawthorne, USA), local background was subtracted, and the data were normalized and 2log transformed. Additional information on barcode screens can be found at http://www.screenic.nki.nl/.

### Gene expression analysis

RNA from mammosphere MCF7 cell cultures, MCF7 cells with overexpression of IC-NOTCH and MCF7 cells with FOXO3A knockdown was isolated by Trizol. Microarray slides were prepared at the central microarray facility (CMF) at the Netherlands Cancer Institute. Sequence-verified cDNA clones (Invitrogen, Huntsville, USA) were spotted onto poly-l-lysine-coated glass slides using the Microgrid II arrayer (Apogent, Cambridge, United Kingdom) with a complexity of 19 200 spots/slide. A complete list of genes and controls included on the slides is available on the CMF Web site (http://microarrays.nki.nl/download/protocols.html), as well as details on the process of preparing the DNA for spotting and preparation of the slides. Fluorescent intensities were normalized and corrected for a variety of biases that affect the intensity measurements.

### Differentiation of HC11 cells

HC11 cells stably transfected with (Figure 1D) or without (Figure 4E) a β-casein-luciferase construct (HC11lux) [55] were cultured in RPMI 1640 medium containing 10% fetal calf serum, L-glutamine, 5 μg/ml Insulin and 10 ng/ml epidermal growth factor (EGF). Cells were washed with PBS to remove the EGF and cells were treated with dexamethasone (1 μM) and the lactogenic hormone prolactin (5 μg/ml) (Sigma) for four days. The HC11lux cells were harvested and measured for luciferase activity. Luciferase assays were performed (Luciferase Assay Kit; Promega, Madison, USA) according to the manufacturer's instructions.

### Determining side population

MCF7 cells (1×10^6^ cells/ml) were stained with 5 μg/ml Hoechst 33342 dye (Molecular Probes, Eugene, USA) and incubated at 37°C for 2 hours. After Hoechst staining, cells were washed and resuspended into 200 ml of cold PBS/2% FCS. Cells were kept on ice until fluorescence activated cell sorting (FACS) analysis. Data acquisition was performed using a FACSCantoII (equipped with red, blue and ultra violet lasers) from BD Biosciences; analysis was performed using FACSDiva software (BD Biosciences). The Hoechst dye was excited with a 405-nm violet laser and detected with 450/BP50 optical filters, respectively.

### Flow cytometry

Cells were incubated for 30 minutes at room temperature with phycoerythrin (PE), or Fluorescein isothiocyanate (FITC) labeled antibodies including PE-conjugated mouse anti-human CD24 (ML5) (BD Pharmingen), PE-conjugated anti-CD133/2 (293C3, Miltenyi Biotec) and FITC-conjugated anti-CD44 (IM7, BD Pharmingen). After antibody staining, cells were washed with icecold PBS/0.1% Human Serum Albumin (HSA), resuspended in 250 μl cold PBS/0.1%HSA. Labeled samples were analyzed using a FACS CantoII (BD Biosciences). Analysis was performed using FACS Diva software (BD Biosciences).

### Antibodies and western blotting

Cells were lysed in the presence of 50mM Tris, pH 7.5, 150 mM NaCl, 1% NP40, 1 complete cocktail of protease inhibitor and deoxycholate and SDS. Cell lysates were separated by 10% SDS-PAGE and transferred to PVDF membranes. Membranes were incubated with anti-phospho AKT (Ser472, #9271, Cell Signaling, Beverly, MA), anti-AKT1/2 (H136 Santa Cruz Biotechnology, Santa Cruz, CA) or anti-FOXO3A (Santa Cruz). Immunoblotting was performed according to the antibody manufacturer's recommenations using enhanced chemiluminescence.

### Limiting dilution breast cancer xenograft mouse model

Animal experiments conformed to the UK Home Office Regulations (Animal Scientific Procedures Act 1986; Project License PPL40/3645). All mice had free access to water, environmental enrichment and a maintenance diet in a 12-hour light/dark cycle at 22°C and 55% humidity. 5 week old female NSG (NOD.Cg-Prkdcscid Il2rgtm1Wjl/SzJ) mice (Charles Rivers Ltd. UK) were implanted with 0.36mg 17β-estradiol 90 day release pellets (Innovative Research of America) one week prior to cell injections. Control and FoxO3a(A3)-transduced MCF7 cells were serially diluted in 0.2mls 50/50 mammosphere medium/matrigel (Cat. 356230, BD Ltd. UK) before sub-cutaneous injection into opposing flanks of NSG mice. Tumor growth was monitored every 3 days and tumor volume was calculated using (Length × Width^2^)/2. At 52 days, a tumor volume of >100mm3 was used as evidence of tumor initiation. CSC frequency was calculated using the L-Calc software (http://www.stemcell.com/en/Products/All-Products/LCalc-Software.aspx).

### Histochemistry

The tumor tissue microarray (TMA) was obtained from the Western Australian Research Tissue Network (WARTN) as previously described [56] and the study was approved by South Manchester Research Ethics committee (05/Q1403/159). Staining was performed using rabbit anti-FKHRL1 (H-144) antibody (sc-11351: Santa Cruz Biotechnology) at a 1/150 dilution and the Envision Kit (DAKO). Nuclear and cytoplasmic compartments of the cells were assessed under high-power. Intensity was scored as 0 (negative), 1 (weak), 2 (moderate), and 3 (strong); percentage of positive cells examined was scored as 0 (negative), 1 (1- 10%), 2 (11-33%), 3 (34-66%), and 4 (66-99%) 5 (100%), and the scores for intensity and proportion combined into a modified H Score using the Allred method [Allred et al., 1998].

### Prognostic value of FOXO3A expression

Time-to-event curves were estimated using the Kaplan-Meier method and compared with the logrank test. Stratified log-rank tests were used to assess the prognostic value of FOXO3A after adjustment for ER and lymph node status, respectively. Cox regression analysis was used to assess the prognostic value of FOXO3A after adjustment for seven commonly used prognostic factors.

## SUPPLEMENTARY FIGURES AND TABLES




















