# Supplementary material for: An integrated genomic approach identifies that the PI3K/AKT/FOXO pathway is involved in breast cancer tumor initiation
Source: Oncotarget. 2015 Nov 22;7(3):2596–610. doi: 10.18632/oncotarget.6354 (PMC4823058; doi:10.18632/oncotarget.6354)
Supplement: Supplementary file 1 [file oncotarget-07-2596-s001.pdf]

An integrated genomic approach identifies that the PI3K/AKT/FOXO pathway is involved in breast cancer tumor initiation

Supplementary Material

Supplemental Table 1

Top 36 shRNAs enriched within the three rounds of mammosphere culturing. Red genes indicate the shRNAs that are enriched in each round of subsequent mammosphere culturing. 381 genes are significantly downregulated in the first round of mammosphere culturing. From these genes five were also present in the shRNA screen (dark blue). 541 genes were significantly downregulated in the second round of mammosphere culturing. From these genes three genes were present in the shRNA screen (light blue).

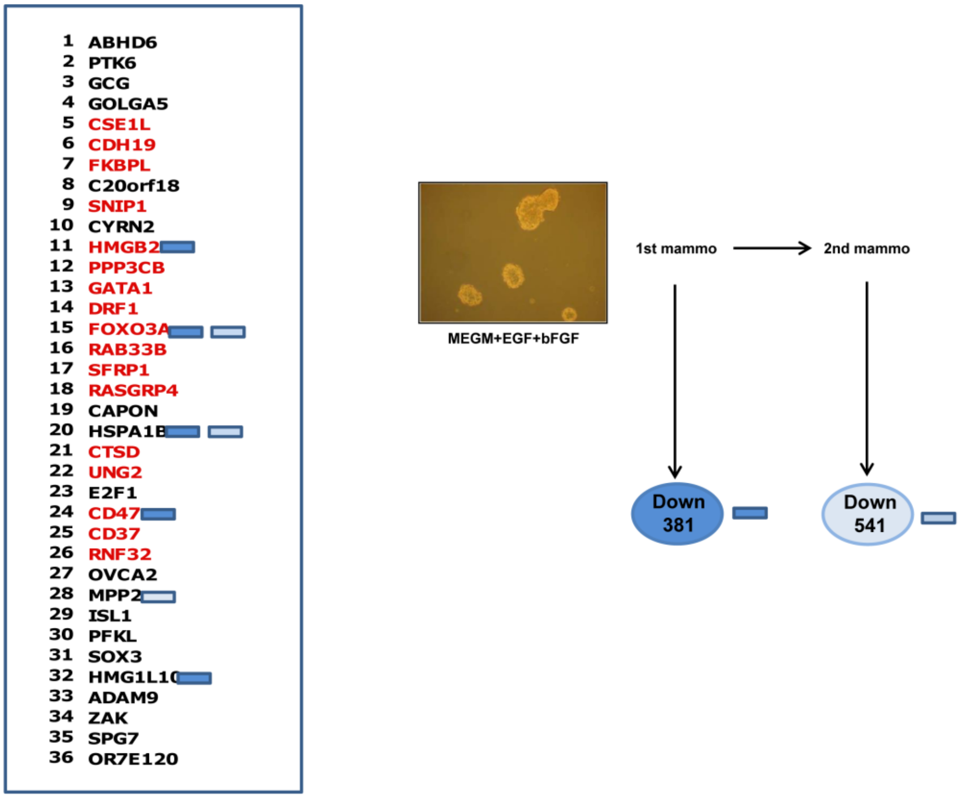

Supplemental Table 2: The gene expression profile of 1st and 2nd round of mammosphere cultures.

(A) Expression profile of 1st round MCF7 mammospheres as compared to attached MCF7 cell cultures (B) Keratins are downregulated in the 1st round of mammosphere culture of MCF7 cells. (C) Expression profile of the 2nd round of MCF mammosphere cultures as compared to attached MCF7 cell cultures (D) Keratins are downregulated in the 2nd round of mammosphere culture of MCF7 cells.

Supplemental Table 2B

Keratin	M	A	P
KRT12	-1.817	12.6545	3.16E-12
KRT16	-0.90517	10.8767	1.45E-05
KRT18	-1.28892	14.4482	0.000165
KRT19	-1.22702	14.3278	2.76E-08
KRT8	-1.43963	14.3618	7.21E-05
KRT8	-1.3242	14.1818	1.20E-05
KRTHB6	-1.44798	13.9597	2.79E-05

Supplemental Table 2D

	M	A	P
KRT14	-0.77269	10.9519	0.007456
KRT15	-0.90838	10.5318	0.014641
KRT19	-1.33987	12.6909	8.88E-07
KRT6A	-0.63638	7.84525	0.025996
KRT8	-1.42711	11.4051	2.87E-09
KRT8	-0.9467	11.5304	0.000128

Supplemental Table 3: The expression profile of MCF7 cells transduced with IC-NOTCH-GFP.

Expression profile of MCF7 cells transduced with control plasmid compared with the expression profile of MCF cells transduced with IC-NOTCH plasmid. 38 genes are downregulated in MCF7 cells with activated NOTCH signaling.

Supplemental Table 4: The gene expression profile of MCF10A cells with knockdown of FOXO3A. (A) Gene expression of MCF10A cells transduced with two shRNAs (FOXO3A-oligo2 and FOXO3A-oligo3) against FOXO3A were compared to the gene expression of cells transduced with the control shRNA (PRS-GFP). (A) Top upregulated genes and (B) Top downregulated genes. (C) Gene Ontology analysis by DAVID of both the up- and down-regulated genes by FOXO3A knockdown.

Supplemental Table 5: The gene expression profile of MCF7 cells with knockdown of FOXO3A. (A) Gene expression of MCF7 cells transduced with two shRNAs (FOXO3A-oligo2 and FOXO3A-oligo3) against FOXO3A were compared to the gene expression of cells transduced with the control shRNA (PRS-GFP). (A) Top upregulated genes and (B) Top downregulated genes. (C) Gene Ontology analysis by DAVID of both the up- and down-regulated genes by FOXO3A knockdown.

Supplemental Table 6: Composition of the breast tumor cohort immunostained for FOXO3A in terms of common prognostic characteristics.

Variable	Patient numbers (%)
**Total**	317 (100)
**Age, years**	
<40	17 (5)
40-49	64 (20)
50—59	86 (27)
≥60	150 (47)
**Tumour Size, mm**	
≤20	165 (52)
21-50	138 (44)
>50	14 (4)
**Positive lymph nodes**	
0	214 (68)
1-3	70 (22)
≥4	33 (10)
**Grade**	
1	81 (26)
2	154 (49)
3	82 (26)
**ER status**	
positive	60 (19)
negative	256 (81)
Missing	1
**HER2 status**	
positive	23 (7)
negative	284 (90)
Missing	10 (3)

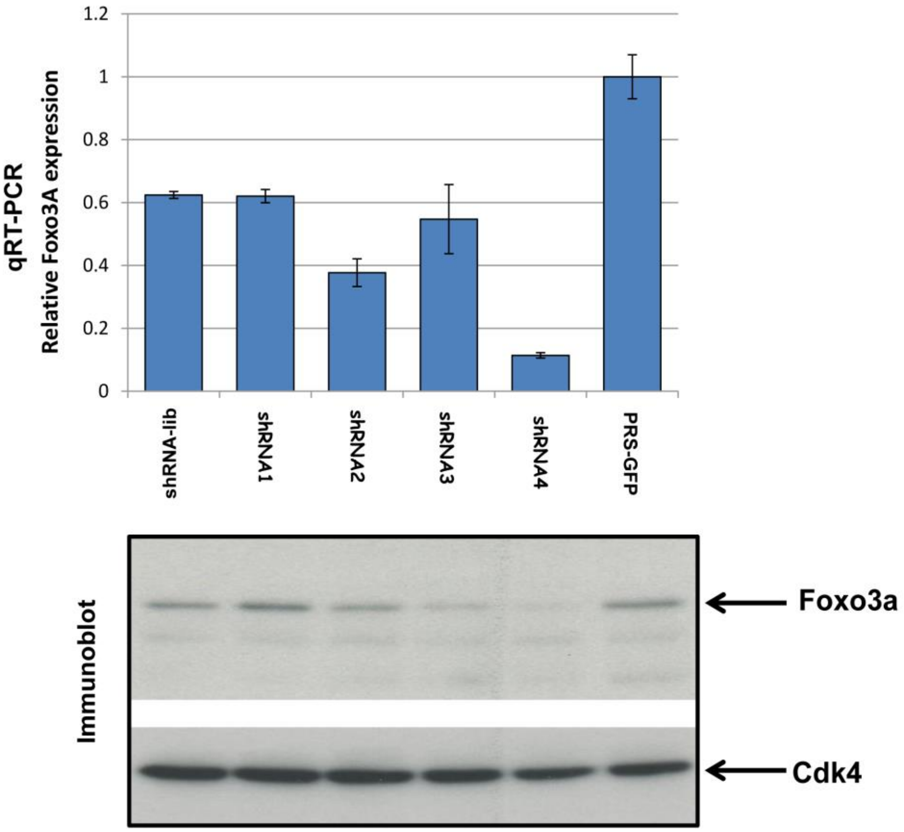

Supplemental Figure 1: Knockdown of FOXO3A by shRNAs in MCF7 cells

pRetrosuper plasmids containing shRNAs against FOXO3A were transduced in MCF7 cells and cells containing the plasmids were selected by puromycin (2□g/ml). The downregulaton of FOXO3A was measured by QRT-PCR (upper panel) and by westernblotting (lower panel) for FOXO3A. In the QRT-PCR the expression of GAPDH is used as a housekeeping gene control and in the western blotting the level of CDK4 is used as the loading control.

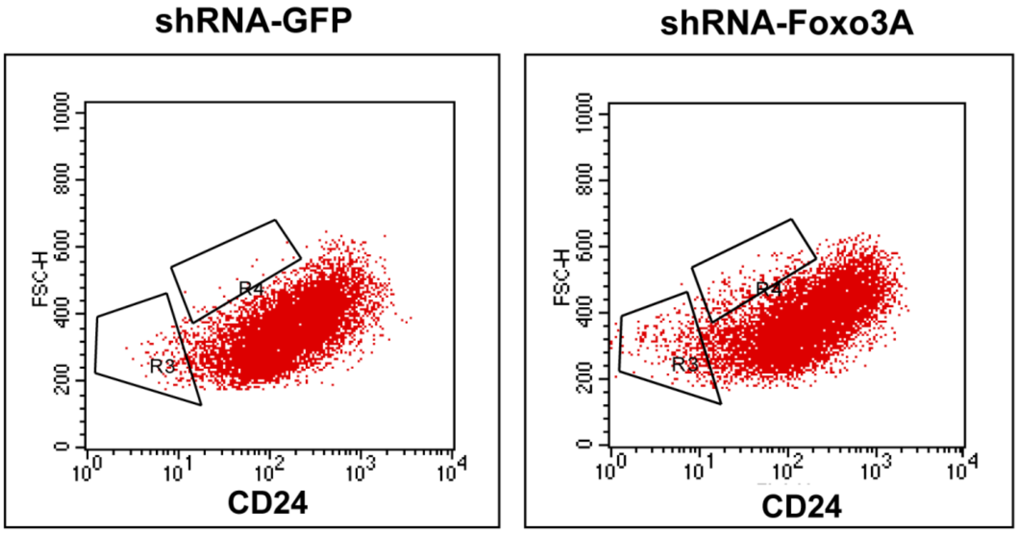

	GFP	shFoxo3A
CD24min (R3)	1.07%	3.15%
CD24low (R4)	0.29%	1,86%

Supplemental Figure 2: Downregulation of FOXO3A results in increased population of CD24low/min MCF7 cells

MCF7 cells were transduced with shRNA-GFP against GFP or shRNA4-FOXO3A against FOXO3A and the expression of CD24 was measured by flow cytometry using anti-CD24-PE.

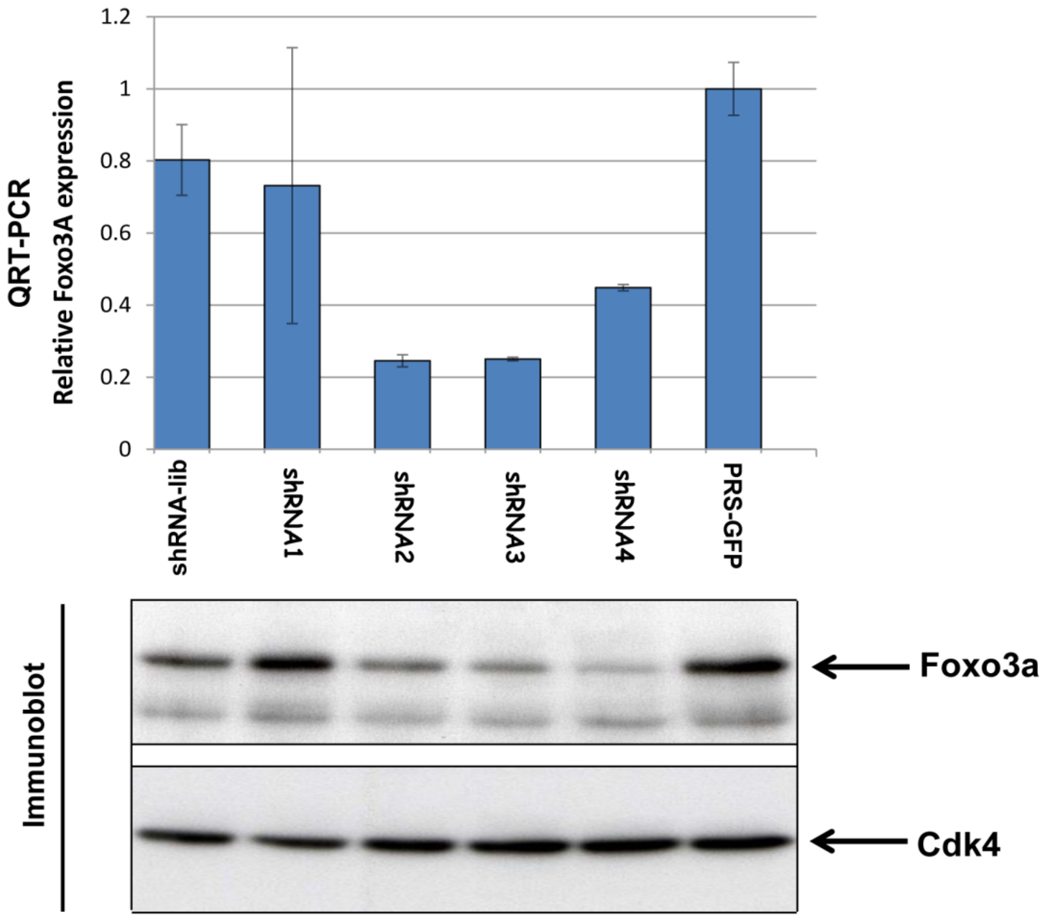

Supplemental Figure 3: Knockdown of FOXO3A by shRNAs in MCF10A cells

pRetrosuper plasmids containing shRNAs against FOXO3A were transduced in MCF10A cells and cells containing the plasmids were selected by puromycin. The downregulaton of FOXO3A was measured by QRT-PCR (upper panel) and by westernblotting (lower panel) for FOXO3A. In the QRT-PCR the expression of GAPDH is used as a housekeeping gene control and in the western blotting the level of CDK4 is used as the loading control.

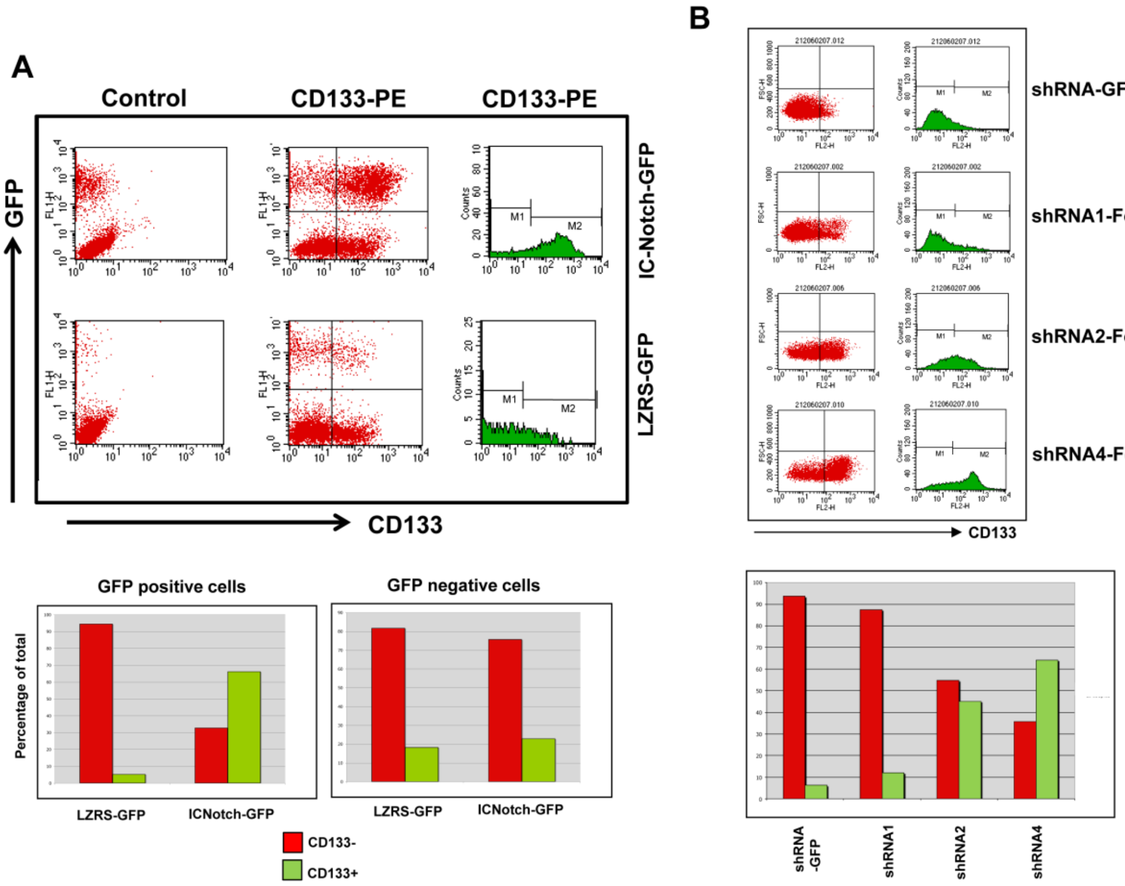

Supplemental Figure 4: The activation of NOTCH and knockdown of FOXO3A results in enhanced CD133 expression.

(A) The low CD133 expressing MCF10A cells were transduced with IC-NOTCH and membrane expression of CD133 was determined in the GFP- and GFP+ population of cells by flow cytometry using anti-CD133-PE. B) MCF10A cells were transduced with shRNAs against FOXO3A (pretrosuper-GFP) and membrane expression of CD133 was determined in the GFP- and GFP+ population of cells by flow cytometry.

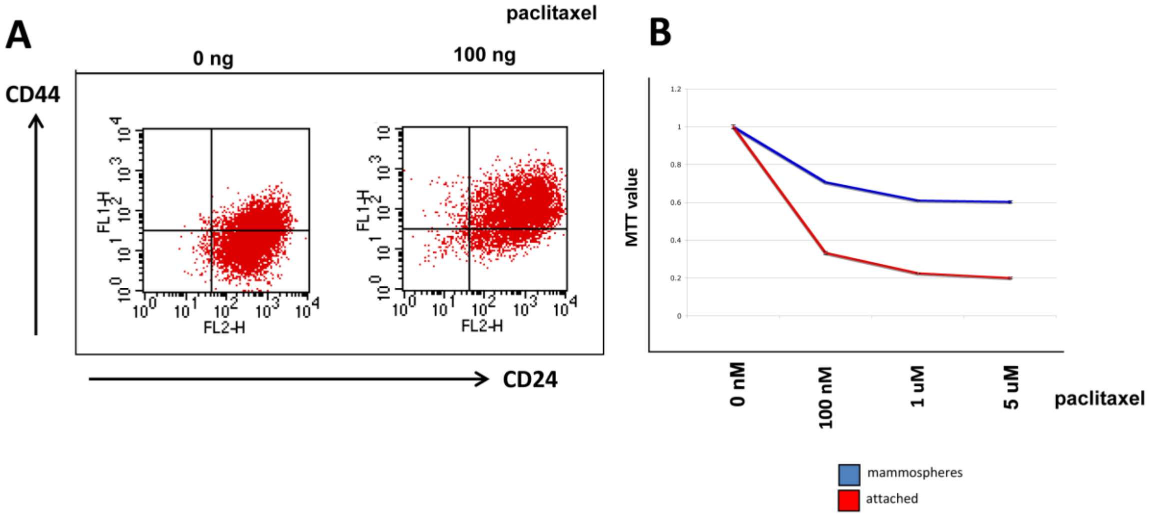

Supplemental Figure 5: Paclitaxel incubation of MCF7 cells results in an increase in the CD44+CD24low population of MCF7 cells. Mammospheres are less sensitive to paclitaxel than MCF7 cells grown under attached conditions. A) MCF cells grown in the presence of paclitaxel (100 ng) and measured for the membrane expression of CD44 and CD24 by flow cytometry. B) MCF7 cells were grown in the presence of paclitaxel under mammosphere and attached culture conditions.

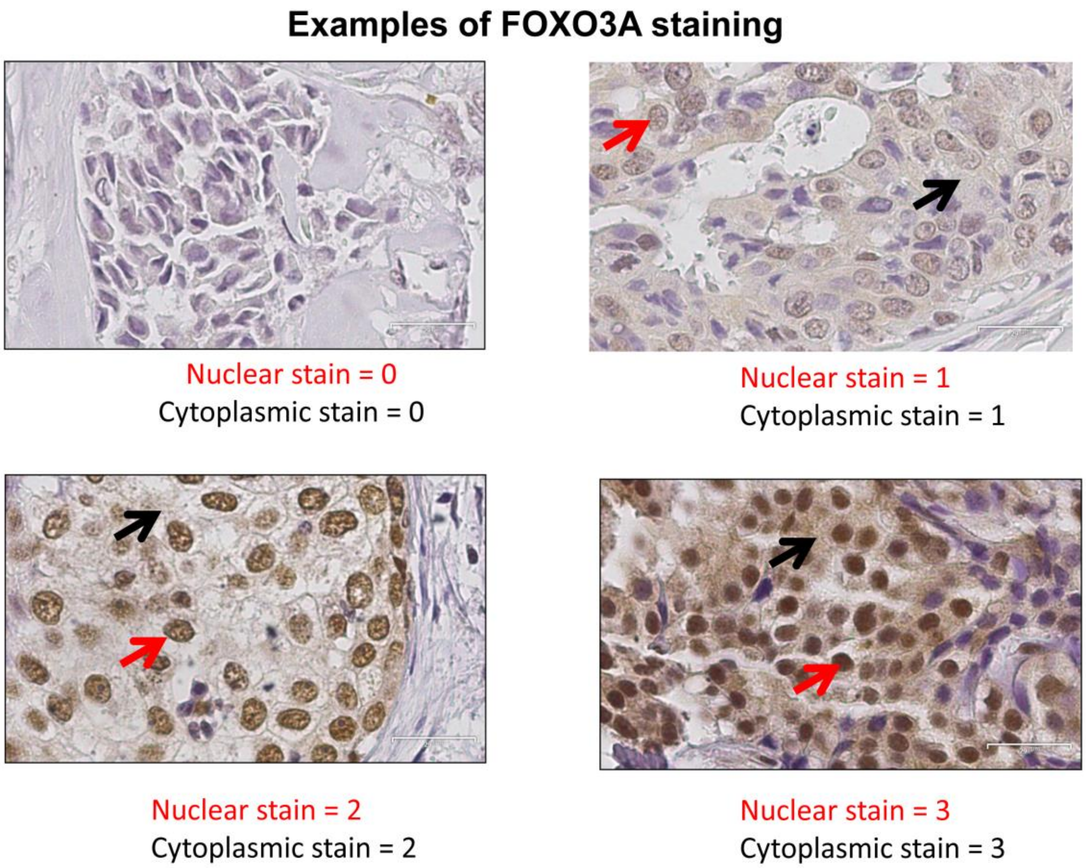

Supplemental Figure 6. Examples of FOXO3A immunostaining intensity: Nuclear and cytoplasmic compartments of the cells were assessed under high-power. Intensity was scored as 0 (negative), 1 (weak), 2 (moderate), and 3 (strong).
